# Removal of Lindane from Aqueous Solution Using Aluminum Hydroxide Nanoparticles with Surface Modification by Anionic Surfactant

**DOI:** 10.3390/polym12040960

**Published:** 2020-04-20

**Authors:** Thi Hang Nguyen, Thi Thuy Linh Nguyen, Tien Duc Pham, Thanh Son Le

**Affiliations:** 1 Faculty of Chemistry, VNU University of Science, Vietnam National University, Hanoi, 19 Le Thanh Tong, Hoan Kiem, Hanoi 100000, Vietnam; hangnt@hau.edu.vn (T.H.N.); nguyenthithuylinh2_t62@hus.edu.vn (T.T.L.N.); 2 Department of Infrastructure and Urban Environmental Engineering, Hanoi Architectural University, Nguyen Trai, Thanh Xuan, Hanoi 100000, Vietnam

**Keywords:** lindane, POPs, adsorption, aluminum hydroxide nanoparticles, SDS

## Abstract

In the present study, we investigated the removal of an emerging pesticide lindane from aqueous solution using synthesized aluminum hydroxide Al(OH)_3_ (bayerite) nanomaterials with surface modification by an anionic surfactant sodium dodecyl sulfate (SDS). The Al(OH)_3_ nanoparticles were characterized by X-ray diffraction (XRD), Fourier transform infrared spectroscopy (FT-IR), scanning electron microscopy (SEM), Brunauer–Emmett–Teller (BET) and zeta potential. The lindane removal using SDS-modified nano-aluminum hydroxide nanoparticles (SMNAH) achieved removal of up to 93.68%, which was 3.3 times higher than that of nano-aluminum hydroxide nanoparticles. The adsorptive removal conditions were studied and found to have an adsorption time of 60 min, a pH of 6, an adsorbent dosage of 25 mg/mL and an ionic strength of 10 mM NaCl. After reusing four times, the removal efficiency of lindane using SMNAH still reached 75%. Two-step adsorption can fit adsorption isotherms of lindane onto SMNAH at two salt concentrations. On the basis of the change in zeta potential, surface functional groups and adsorption isotherms, we suggest that the formation of a bilayer micelle induced the removal of lindane.

## 1. Introduction

Pesticides are important chemicals that are often used in agricultural activities. Nevertheless, most pesticides are harmful and dangerous due to their toxicity and very low self-degradation in nature [1]. Some pesticides belong to the persistent organic pollutants (POPs) group that are synthetic organic substances with some intrinsic chemical–physical characteristics such as persistency, toxicity, bio-accumulation and long transportable nature [2]. POPs can cause many effects, including carcinogenic, teratogenic, immunologic, endocrine and neurological problems in organisms [3]. There are different kinds of POPs, in which organochloride pesticides (OCPs) have been one of the most important with regard to environmental concerns. Among the OCPs, lindane is known to be very persistent, toxic and bio-accumulative in humans and animals [4]. So far, the mitigation of different environmental pollutants has been increasingly important in fields such as pharmaceuticals [5], heavy metals [6,7] and dyes [8,9]. Many pollutants have been studied with regard to their removal from aqueous solutions by different techniques.

Various techniques have been studied and developed for lindane and OCP removal or degradation. For instance, adsorption [1,10,11] and photocatalytic degradation [2,12,13] are widely used due to their very high efficiency compared with other methods. While photocatalysis using nanomaterials is a novel method with which to decompose the lindane compounds to less-toxic or non-toxic substances, adsorption is an excellent technique to remove the lindane from aqueous solutions in the presence of a high performance adsorbent with a high specific surface area. Metal oxide nanomaterials, such as titanium oxide and zinc oxide, can be used for both photocatalysis and adsorption. However, the common metal hydroxides, which are dominant compositions of various soils, have no photocatalytic activities. This also implies that metal oxides from soils or clays are much cheaper than photocatalytic materials.

Aluminum hydroxide is non-activated photocatalyst, which is well-known in chemical engineering and related fields. Among many structural phases of aluminum hydroxide, bayerite, α-Al(OH)_3_, has a pillared double layer form that is easily synthesized with a low calcinated temperature [14]. The nanosized α-Al(OH)_3_ has a quite high specific surface area that is very different to α-Al_2_O_3_ [15]. It is evident that the higher the specific surface area, the greater effectiveness of the adsorbent. Nevertheless, α-Al(OH)_3_ has a hydrophilic surface, while the lindane surface is hydrophobic. Therefore, surface modification of α-Al(OH)_3_ is necessary to change the hydrophobicity of the adsorbent. Surface modification by different methods and chemicals is a powerful tool for fine-tuning the properties of various materials for different applications, such as hydroxyapatite [16], polybenzimidazone/polydopamine [17], biophenol coating [18] and corn oil [19]. Surfactants are amphoteric substances that contain both hydrophobic and hydrophilic components. Recently, anionic surfactants, such as sodium dodecyl sulfate-modified aluminum hydroxide, have become well-known adsorbents that are successfully used to enhance removal of various inorganic and organic pollutants [20,21,22,23,24,25]. However, to the best of our knowledge, the removal of lindane through adsorption techniques using synthesized α-Al(OH)_3_ nanoparticles with SDS modification has not been studied.

For adsorption systems, Freundlich, Langmuir and Temkin isotherms are very common models that describe adsorption behavior [26,27,28]. In contrast, these models could not be used for surfactant adsorption. A two-step model proposed by Zhu and co-worker [29] was achieved for the adsorption of surfactants and absorbates onto surfactant-modified adsorbents [9,22,23,25,29,30,31]. Therefore, a two-step model is applicable for lindane adsorption onto synthesized nano-aluminum hydroxide with surface modification by SDS.

This first work aims to study the removal of lindane using SDS-modified nano-aluminum hydroxide (SMNAH). The effective conditions for adsorption of lindane onto SMNB were thoroughly investigated. Adsorption mechanisms of lindane onto SMNAH are suggested based on the changes in zeta potential and vibration groups by Fourier transform infrared spectroscopy (FT-IR), as are adsorption isotherms.

## 2. Materials and Methods

### 2.1. Materials

Sodium hydroxide (NaOH) and aluminum nitrate Al(NO_3_)_3_·9H_2_O, used for synthesizing nano absorbents were delivered from Merck, Singapore.

**Figure 1:**
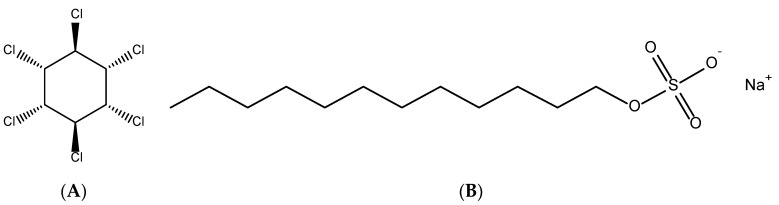
The chemical structures of lindane (**A**) and sodium dodecyl sulfate (SDS) (**B**).

The 1,2,3,4,5,6-hexachlorocyclohexane (γ-isomer, CAS 58-89-9) for gas chromatography (with purity > 99.0%), with a molecular weight of 290.81 g/mol, was purchased from Tokyo Chemical Industry (Tokyo, Japan). Sodium dodecyl sulfate (SDS) (with purity > 95%) delivered from Scharlau (Barcelona, Spain, EU), did not have further treatment. Figure 1 indicates the chemical structures of lindane (A) and SDS (B). The ionic strength was changed by adding NaCl, HCl and NaOH (volumetric analysis grade, Merck, Darmstadt, Germany). 

The solution pH was monitoring by a pH meter (HI 2215, Hanna, Woonsocket city, RI, USA). Three standard buffers of 4.01, 7.01 and 10.01 (Hanna) were used to calibrate the pH electrode every use. Other analytical grade chemicals used for gas chromatography were purchased from Merck. Ultrapure water with a resistivity of 18.2 MΩ.cm was produced from an ultrapure water system (Labconco, Kansai City, MO, USA).

### 2.2. Synthesis of Aluminum Hydroxide Nanoparticles

Aluminum hydroxide nanoparticles were synthesized by following the proposed procedure with a minor modification [32]. Firstly, to prepare the 4 M NaOH and 1 M Al(NO_3_)_3_ solutions, the appropriate amounts of NaOH pellets and Al(NO_3_)_3_ were dissolved in ultrapure water. Aluminum hydroxide was received by titrating 1 M Al(NO_3_)_3_ with 4 M NaOH in a plastic barker with continuing magnetic stirring. The stirring speed of 500 rpm was kept constant during the titration. When the white wet precipitation was obtained, it was centrifuged at 6000 rpm (Digisytem, Taiwan) to separate the solid and liquid. Then, the material was dried at 80 °C for 24 h. The white powders were then heated at 600 °C for 12 h (sample A) and at 800 °C for 6 h (sample B) in a Nabertherm furnace before reducing the temperature to 40 °C in a desiccator. Finally, the aluminum hydroxide nanoparticles were kept in a PE bottle.

### 2.3. Characterization Methods

The synthesized aluminum hydroxide was evaluated by different physiochemical techniques, including X-ray diffraction (XRD), scanning electron microscopy (SEM), Fourier transform infrared spectroscopy (FT-IR) and ζ potential.

The XRD patterns were obtained on an X-ray diffractometer (Bruker D8 Advance, Karlsruhe, Germany) with CuK*_α_* radiation (*λ* = 1.5418 Å). The XRD spectra were gathered from 10° to 70° (2θ) with an increase of 0.03°. The FT-IR spectra were gathered with an infrared spectrometer (Affinity-1S, Shimadzu, Kyoto, Japan). The FTIR spectra of nano-aluminum hydroxide particles, SDS-modified nano-aluminum hydroxide (SMNAH) and SMNAH after lindane adsorption were obtained with 4 cm^−1^ resolution of at 25 °C and atmospheric pressure.

The particle size distribution of synthesized nano-aluminum hydroxide was evaluated by SEM (Hitachi S4800, Tokyo, Japan). ImageJ software was used to calculate the mean size of nano-aluminum hydroxide particles.

The BET method was used to determine the specific surface area of the adsorbent by using the surface area analyzer Micromerities (TriStar 3000, Norcross, GA, USA). The adsorption and desorption isotherm of nitrogen (N_2_) were carried out in a 9 mL cell with an outgas condition of 150 °C in 90 min.

The surface charge of synthesized nano-aluminum hydroxide at different pH values, and the surface charge of SMNHA and SMNHA after lindane adsorption at pH 6 (0.01M NaCl) were determined by using Zetasizer Nano ZS (Malvern, Worcestershire, UK). 

The zeta (ζ) potential from electrophoretic mobility was calculated by using Smoluchowski’s equation [33].

(1) $$\zeta =\frac{u_{e}\eta }{\varepsilon_{rs}\varepsilon_{0}}$$

where ζ is the ζ potential (mV), *u_e_* is the electrophoretic mobility (µm cm/sV), *η* is the dynamic viscosity of the liquid (mPa·s), *ε_rs_* is the relative permittivity constant of the electrolyte solution and *ε*_0_ is the electric permittivity of the vacuum (8.854 × 10^−12^ F/m).

### 2.4. The Analysis of Lindane

The lindane in the aqueous solution was extracted by n-hexane. The extracts were concentrated and water was removed by anhydrous sodium sulfate. After that, the extracts were quantified by gas chromatography with an electron capture detector (GC-ECD) using a Scion 456 GC (Scion Instruments, New York, NY, USA) coupled with CP-8400 Autosampler (Bruker, Kuala Lumpur, Malaysia). To separate lindane in the GC system, a capillary column (DB-5, a 5% phenyl 95% methyl polysiloxane phase, 30 m × 0.25 mm × 0.25 μm (Agilent Technologies, Palo Alto, CA, USA) was used. The parameters of GC-ECD for lindane determination are indicated in Table 1.

### 2.5. Adsorption Studies

The adsorption was carried out using the batch technique in 15 mL Falcon tubes at 25 ± 2 °C. All adsorption experiments were performed in triplicates.

The SDS adsorption onto synthesized nano-aluminum hydroxide to form SDS-modified nano-alumina (SMNAH) was conducted with 0.01 M SDS concentrations at pH 4 for 2 h. Then, SDS desorption was conducted with various times of H_2_O washing and decantation using a centrifuge. Detail of the surface modification can be found in our previously published papers [22,30]. 

For lindane removal by the adsorption technique, various adsorbent dosages were mixed with 10 mL lindane in acetonitrile (ACN)/water in the presence of different NaCl concentrations. The effective conditions (contact time, pH, adsorbent dosage and ionic strength) of lindane removal were investigated. The lindane in the aqueous solution was quantified by the procedure in Section 2.4.

The adsorption capacities of lindane onto SMNAH were calculated by Equation (2).

(2) $$\Gamma =\frac{Ci-Ce}{m}\times 1000$$

where *Ci* and *Ce* are the initial concentration and the equilibrium concentrations of lindane (µg/L), respectively; *m* is the adsorbent dosage (g/L); and *Γ* is the lindane adsorption capacity (µg/g).

The removal (%) of lindane was determined by the following Equation.

(3) $$Removal\left(\%\right)=\frac{C_{i}-C_{e}}{C_{i}}\times 100\%$$

where *C_i_* is the initial concentration (µg/L) and *C_e_* is equilibrium concentration of lindane (µg/L).

The adsorption isotherms lindane onto SMNAH were fitted by the general isotherm equation. The general isotherm equation [29] is:

(4) $$\Gamma =\frac{\Gamma_{\infty }k_{1}C\left(\frac{1}{n}+k_{2}C^{n-1}\right)}{1+k_{1}C\left(1+k_{2}C^{n-1}\right)}$$

where *C* is the equilibrium concentration of lindane; Γ is the amount of adsorbed lindane at concentration *C*; $\Gamma_{\infty }$ is the maximum adsorption at high concentrations; $k_{1}$ and $k_{2}$ are equilibrium factors involved in the first and second step, respectively; and n is the number of clusters of the adsorption layer. 

## 3. Results and Discussion

### 3.1. Characterization of Synthesized Aluminum Hydroxide Nanoparticles

**Figure 2:**
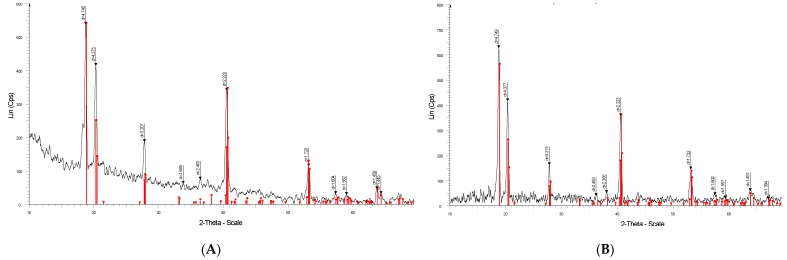
XRD patterns of synthesized α-Al(OH)_3_ (bayerite) samples (**A**) and (**B**).

The XRD patterns of two samples (A) and (B) of synthesized aluminum hydroxide nanoparticles are indicated in Figure 2. Although the intensities and the signals of the two XRD patterns of samples A and B are slightly different due to the temperature and time for calcination, the main peaks of the structural phases are the same. The sharp peaks appeared with high intensity at 2θ = 18.8°, 20.5°, 27.9°, 40.7° and 53.2° showing the crystalline of bayerite with high purity [34].

**Figure 3:**
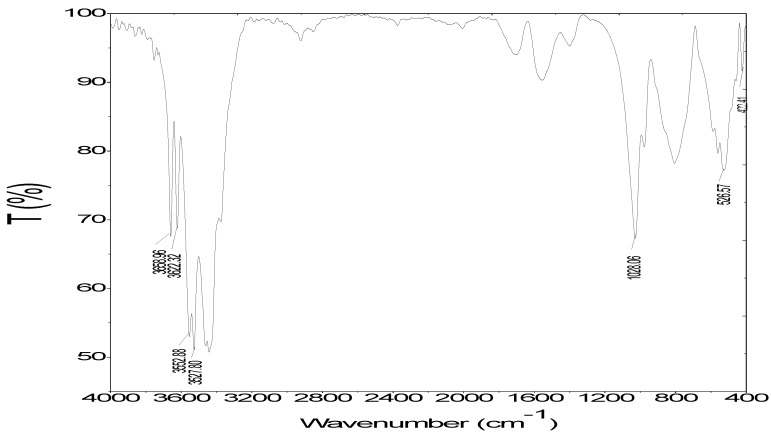
The FT-IR spectra of synthesized nano-aluminum hydroxide nanoparticles.

Figure 3 shows that some sharp peaks in the FT-IR spectra of the α-Al(OH)_3_ (bayerite) appeared at 3658.96 and 3622.32 cm^−1^, and high intensity peaks were at 3552.88 and 3527.80 cm^−1^. These are assigned to the –OH stretching vibration in the aluminum hydroxide structure. The peaks appeared at 1028.06, 526.57 and 422.41 cm^−1^, were assigned to vibration of Al–O bending [35]. The broader peaks at 806.25 cm^−1^ and 526.57 cm^−1^ also indicated the vibration of Al–O bending [36].

**Figure 4:**
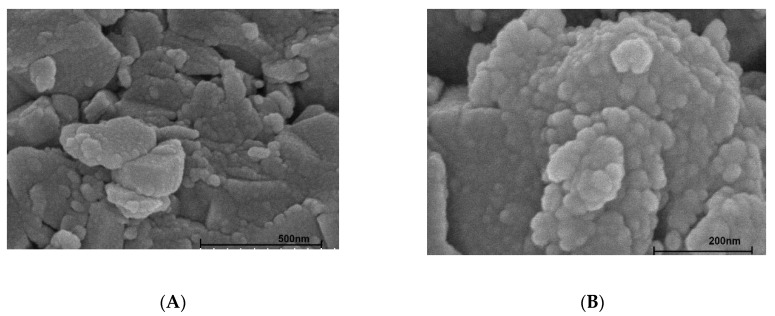
SEM images of nano-aluminum hydroxide with different scales: 500 nm (**A**) and 200 nm (**B**).

The SEM images of nano-aluminum hydroxide (NAH) in Figure 4 indicates the sphere particles of synthesized material. Using the SEM images, we obtained an average diameter of about 30 nm for NAH particles. The average diameter by SEM is in good agreement with the mean particle sizes of NAH by Scherer’s equation [37], using the above XRD patterns in which an average diameter of particles was found to be 28 ± 5 nm.

**Figure 5:**
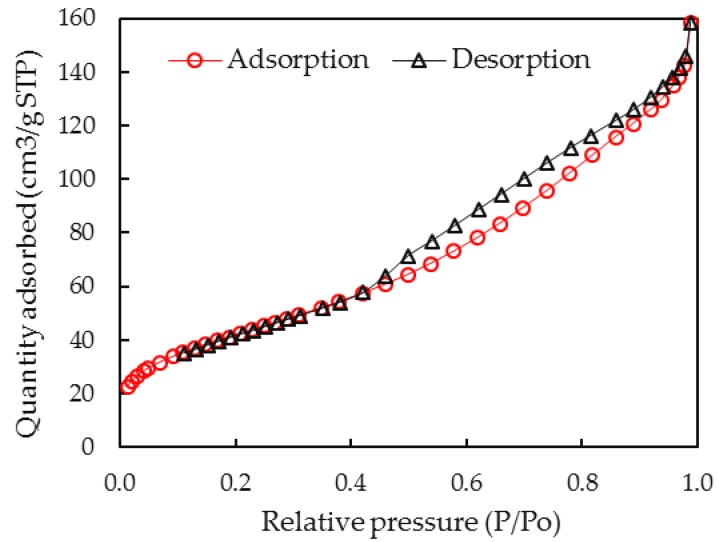
Adsorption isotherm of N_2_ onto synthesized nano-aluminum hydroxide.

The specific surface area of the NAH by BET method was calculated from N_2_ adsorption-desorption isotherms. Based on the adsorption and desorption of N_2_ onto NAH in Figure 5, the specific surface area was found to be about 154.4 m^2^/g.

The specific surface area of synthesized NAH is quite high, which is good for adsorption. Nevertheless, to enhance lindane removal using NAH, the surface charge modification is needed because lindane is a much bigger molecule than N_2_.

**Figure 6:**
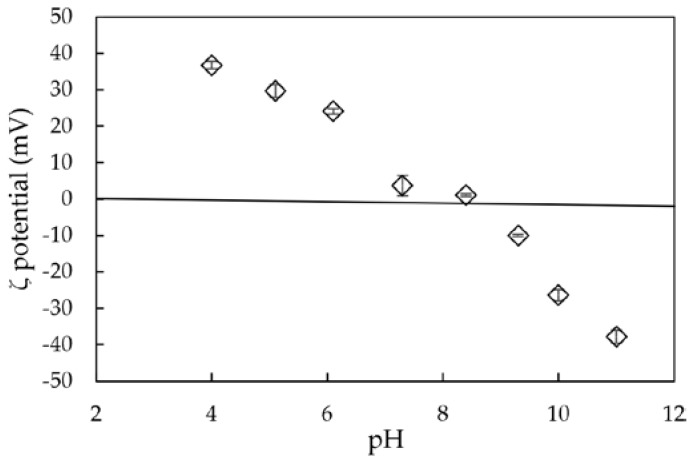
The ζ potential of nano-aluminum hydroxide at different pH levels in 10 mM NaCl.

Figure 6 shows the ζ potential of NAH in the pH range of 4–11 in 10 mM NaCl. The charging property of NAH was highly dependent on the pH. The point of zero charge (PZC) was found to be about 9.0, which was similar to previous studies [15,34]. 

The above results show that the NAH-containing bayerite phase was successfully fabricated with surface functional groups and strong hydrophilic behavior, except for neutral and weak basic media. Therefore, to increase the lindane removal, a surface modification to change the hydrophobic property is necessary. To achieve this purpose, sodium dodecyl sulfate (SDS) as a strong surfactant was selected to modify the NAH surface.

### 3.2. Comparison of the Removal of Lindane Using Nano-Aluminum Hydroxide without and with SDS

**Figure 7:**
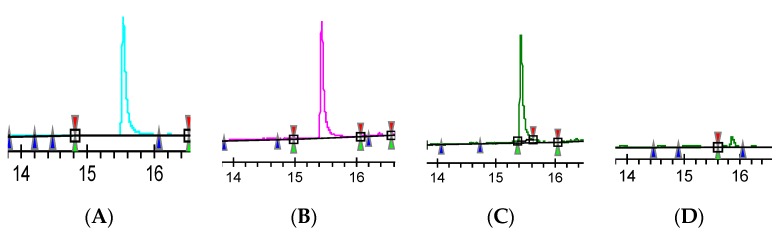
The GC-ECD chromatograms for determination of 200 µg/L lindane (**A**), without adsorption in the presence of SDS (**B**), after adsorption onto synthesized nano-aluminum hydroxide (NAH) (**C**) and after adsorption onto SDS-modified nano-aluminum hydroxide (SMNAH) (**D**).

The synthesized nano-aluminum hydroxide (NAH) was modified by SDS at pH < PZC to put a high number of SDS molecules onto the NAH surface [38]. With an initial concentration of 0.01 M, SDS was greater than the CMC (critical micelle concentration), and the bilayers of admicelles were completely formed [30]. As a result, the SDS modified nano-aluminum hydroxide (SMNAH) was formed. To emphasize the importance of SDS adsorption onto NAH as a novel adsorbent for lindane removal, we compared the chromatograms for the determination of 200 µg/L lindane with different conditions shown in Figure 7. As can be seen, the decreases in peak areas at the lindane retention time of about 15.75 min were not significant when using only SDS (Figure 7B) or only NAH (Figure 7C). On the other hand, only very small peaks appeared in Figure 7D, indicating that a very high removal efficiency of lindane was obtained when using SMNAH. 

**Figure 8:**
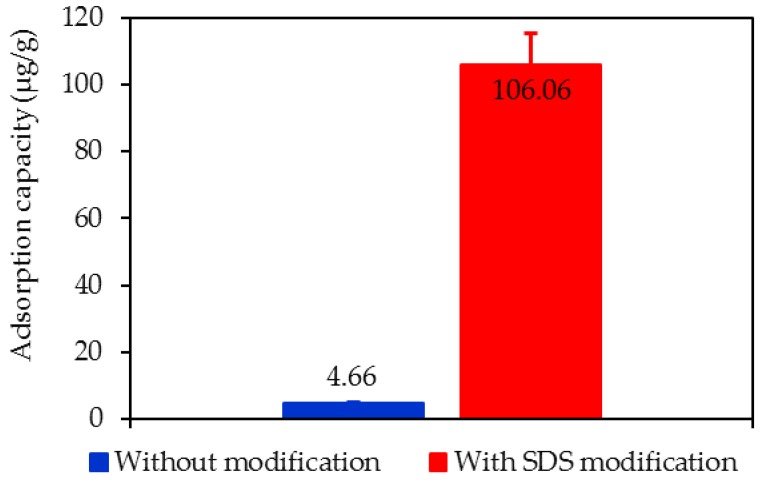
Adsorption capacity of lindane using synthesized nano-aluminum hydroxide (NAH) without and with SDS modification (*C_i_* (lindane) = 1500 µg/L, pH 6, contact time 60 min, adsorbent dosage 25 mg/mL). Error bars indicate the standard deviations of three replicates.

Figure 8 shows the adsorption capacity of lindane using NAH and SMNAH using Equation (2) with an initial concentration of Lindane (Ci) = 1500 µg/L, while other experimental conditions were fixed, corresponding to the case without and with SDS modification, respectively. As can be seen in Figure 8, the adsorption capacity of lindane increased dramatically up to 22.8 times from 4.66 to 106.06 µg/g with the initial lindane concentration of 1500 µg/L. This trend is similar to the removal of organic pollutants using surfactant-modified alumina (SMA). For the lindane removal, an increase of 3.3 times was obtained when using SMNAH. Due to the much higher removal and adsorption capacity of lindane using SMNAH compared with NAH, the below section only optimizes some effective conditions for lindane removal using SMNAH.

### 3.3. Adsorptive Removal of Lindane Using Surfactant-Modified Nano-aluminum Hydroxide (SMNAH)

There are four important parameters that influence the adsorptive removal of lindane using SMNAH, including contact time, solution pH, adsorbent dosage and ionic strength. The effects of these parameters are now systematically studied and discussed in detail.

**Figure 9:**
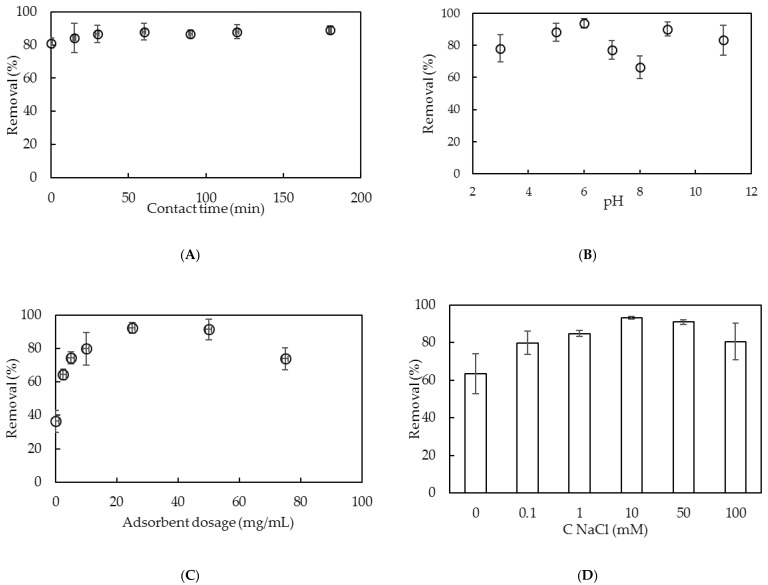
The effects of contact time (**A**), pH (**B**), adsorbent dosage (**C**) and ionic strength (**D**) on lindane removal using surfactant-modified nano-aluminum hydroxide (SMNAH) (*C_i_* (lindane) = 200 µg/L. Error bars indicate the standard deviations of three replicates.

For the adsorption study, contact time is an important factor because it strongly influences the equilibrium process. The effect of contact time on the lindane removal of SMNAH was tested in the range of 0–180 min. Figure 9A shows that the lindane adsorption reached an equilibration at 60 min, where the removal was about 90%. This is much faster than lindane removal using a fungal biosorbent, which requires more than 210 min [10]. The contact time of 60 min was kept for further studies of effective conditions on lindane removal using SMNAH.

The solution pH plays an important role in the lindane removal because the desorption of SDS is strongly influenced by the change of pH [24,30]. The effects of pH on the lindane removal using SMNAH were carried out from pH 3 to 11 in 1 mM NaCl (Figure 9B). As can be seen in Figure 9B, lindane removal achieved its maxima at two pH values of 6 and 9. At pH 6, the bilayers of SDS admicelles may be dominant [39], while the SDS desorption is negligible [24]. On the other hand, at pH 9 the net charge of the adsorbent is close to zero due to the pH ≈ PZC. At pH 9, lindane is a strong hydrophobic compound that easily contacts the surface by non-electrostatic, rather than electrostatic, interaction. It is clear that the lindane removal at pH 6 achieved the highest efficiency, while the error bar indicating the standard deviation of triplicates is the smallest. On the other hand, pH 6 is the best solution condition for lindane removal. Thus, pH 6 was selected for lindane removal using SMNAH.

The adsorbent dosage influences the number of linking sites and specific surface area of the adsorbent [40]. The amounts of SMNAH were changed from 0 to 75mg /mL (Figure 9C). As can be seen in Figure 9C, the lindane removal using SMNAH increased with increasing the adsorbent dosage from 0 to 25 mg/mL due to the increase in total charge density and binding sites. However, a high amount of adsorbent may induce the fast flocculation of nanoparticles [41,42]. Therefore, the optimum adsorbent dosage was 25 mg/mL.

Electrostatic interaction is influenced by ionic strength because an increase in salt concentration can cause a decrease in the electrostatic force. Lindane removal using SMNAH was conducted at different NaCl concentrations, ranging between 0 and 100 mM (Figure 9D).

Figure 9D shows that the lindane removal grew significantly with an increase in ionic strength from 0 to 10 mM. This suggests a hydrophobic interaction between the non-polar lindane molecular and alkyl core of SDS bilayer admicelles. However, the lindane removal declined when increasing the ionic strength from 10 to 100 mM due to the SDS desorption on the alumina surface [25]. The influence of ionic strength is studied further in the section on the adsorption isotherms presented below.

### 3.4. Adsorption Isotherms of Lindane on SDS-Modified Nano-Aluminum Hydroxide (SMNAH)

**Figure 10:**
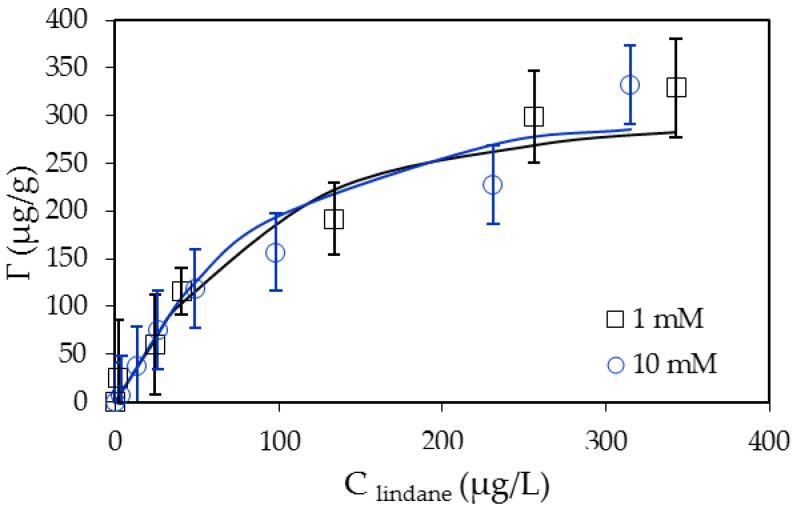
Adsorption isotherms of lindane onto SDS-modified nano-aluminum hydroxide (SMNAH) at two NaCl concentrations. The points are experimental data and solid lines are fitted by a two-step adsorption model. Error bars indicate the standard deviations of three replicates.

The effect of ionic strength on lindane adsorption onto SMNAH was clarified on the isotherms. Figure 10 shows that lindane adsorption was independent of NaCl concentrations. It is evident that high salt concentration induced the decrease of electrostatic interaction between the charged surface and opposite ions. Since lindane is a hydrophobic pesticide, other non-electrostatic interactions, such as hydrophobic interactions, could promote adsorption. This phenomenon is similar to SDS adsorption onto laterite soil at high pH, in which hydrophobic interactions are the main driving force.

Figure 10 also indicates that at two ionic strengths, the experimental results of lindane onto SMNAH can be fitted using the general isotherm equation (Equation (4)) with the fit parameters in Table 2. At two NaCl concentrations of 1 and 10 mM, the maximum adsorption capacities of lindane were insignificant. Interestingly, the same fit parameters (*k*_1_ and *n*) for two isotherms can be applicable while the value of *k*_2_ shows a minor difference. This suggests that *k*_2_ may be a parameter with which to predict a non-electrostatic interaction. The higher the salt concentration is, the higher the hydrophobic interaction and the higher the value of *k*_2_ is.

### 3.5. Adsorption Mechanisms of Lindane onto SDS-Modified Nano-Aluminum Hydroxide (SMNAH)

In this part, the adsorption mechanisms of lindane onto SMNAH are discussed in detail by evaluating the ζ potential change and the vibrational surface group change by FT-IR and lindane isotherms onto SMNAH.

**Figure 11:**
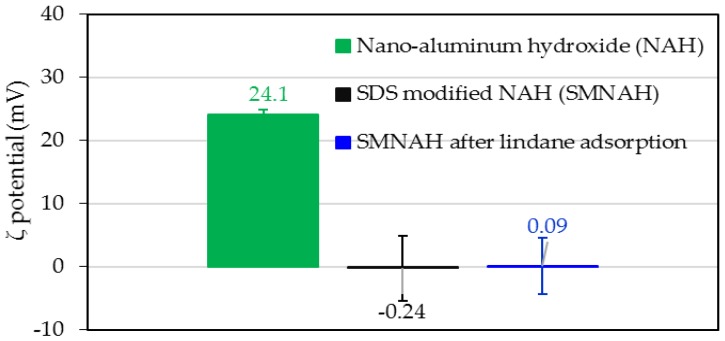
The ζ potential of synthesized nano-aluminum hydroxide (NAH), SDS-modified nano-aluminum hydroxide (SMNAH) and SMNAH after lindane adsorption in 10 mM NaCl (pH 6.0).

Figure 11 indicates that at pH 6, the ζ potential of synthesized nano aluminum hydroxide (NAH) was positive (ζ = + 24.1 mV). Interestingly, after SDS modification at high concentration, the material was sequentially washed to create the SMNAH with a local bilayer formation. As a result, the surface charge of SMNAH was very small and negative with a ζ = −0.24 mV. This result implies that the hydrophobic interaction of the alkyl core in the SDS admicelles was highly promoted [24,30]. However, after lindane adsorption with an increase of hydrophobicity, the net charge of SMNAH changed insignificantly (ζ = +0.09 mV). The changes in ζ potential indicate that lindane adsorption onto SMNAH was probably controlled by hydrophobic interactions, while the electrostatic one was negligible.

**Figure 12:**
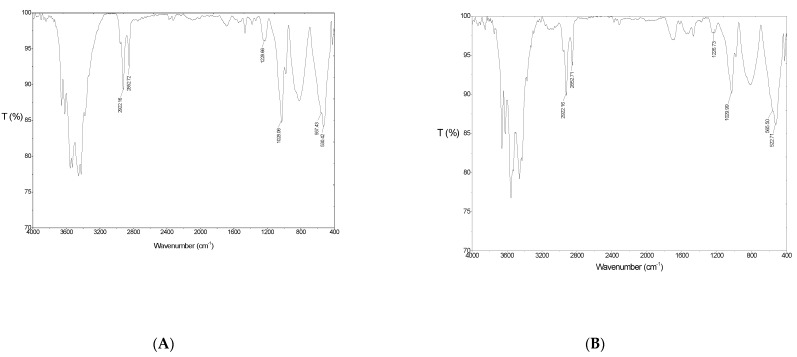
The FT-IR spectra of SMNAH (**A**) and SMNAH after lindane adsorption (**B**).

FT-IR is a good technique with which to predict the changes in vibrational surface groups during adsorption [43]. The FT-IR spectra of SMNAH and SMNAH after lindane adsorption range from 400 to 4000 cm^−1^, as is shown in Figure 12.

Figure 12A shows that the –OH stretching appeared strongly at around 3500 cm^−1^ for SMNAH, which was similar to NAH (Figure 3). However, the sharp peaks of –CH_2_– are asymmetrical, and symmetrical stretching, assigned at 2922.16 and 2852.72 cm^−1^, occurred with high intensity in the FT-IR spectra of SMNAH [44], demonstrating that the hydrophobic interaction took place on the NAH surface. Furthermore, the strong peak for the sulfate group of SDS at about 1226.73 cm^−1^ [24,30] decreased the intensity and shift to a longer wavenumber of 1228.66 cm^−1^ in the spectra of SMNAH, indicating the SDS bilayers and/or admicelles on the surface of NAH after modification.

After lindane adsorption, the boarding peaks at 1028.06 and 557.43 cm^−1^ were assigned for vibration of Al–O bending, and the peaks of alkyl stretching decreased in intensity while the sulfate groups at about 1228 cm^−1^ still occurred. This indicated that that lindane adsorption onto SMNAH was mainly by the interaction between the hydrophobic lindane ring and alkyl chain of bilayer admicelles of SDS on NAH. These results agree well with the zeta potential data and lindane adsorption isotherms in which lindane adsorption onto SMNAH was induced by hydrophobic interactions.

Adsorption isotherms of lindane onto SMNAH were independent of ionic strength, with almost the same fit parameters by the two-step model, which demonstrates that the electrostatic attraction between the lindane molecules and the negative SMNAH surface was negligible. The charging behavior of SMNAH before and after adsorption monitored by ζ potential was in accordance with the change in surface modification by FT-IR spectroscopy. This demonstrates evidence of hydrophobic interactions between the alkyl core of the bilayers of the SDS admicelles and the lindane molecules. Therefore, we suggest that the presence of admicelles with hydrophobic bilayer cores of SDS molecules onto NAH surface promoted lindane adsorption onto SMNAH.

### 3.6. Comparison of Effectiveness of Surfactant-Modified Nano Aluminum Hydroxide (SMNAH) and Other Adsorbents for Lindane Removal

Adsorptive removal of the high hydrophobicity pesticide lindane from aqueous solution is still a big challenge for scientists due to the low adsorption capacity and removal efficiency. The synthesized nano aluminum hydroxide with surface modification by SDS in the present study is a new adsorbent to the best of our knowledge. When using SMNAH, the adsorption capacity and the removal efficiency were 325 µg/g and 93.68%, respectively, under the optimum adsorptive conditions. We found that the SMNAH investigated in this work achieved the highest removal efficiency and the biggest adsorption capacity compared to many kinds of adsorbents for lindane removal (Table 3).

**Figure 13:**
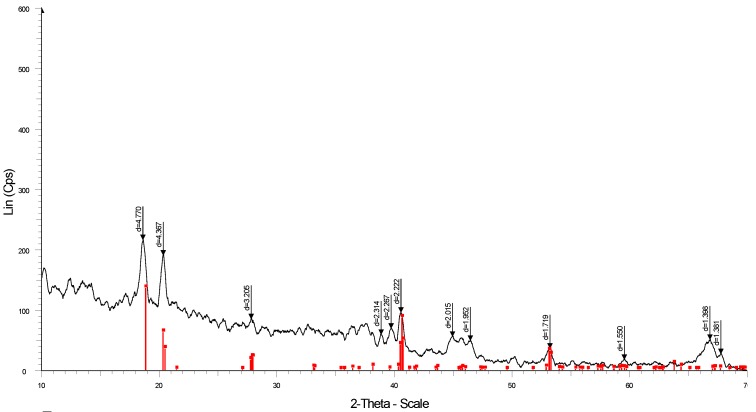
XRD pattern of nano aluminum hydroxide (NAH) after lindane adsorption and SDS modification.

All adsorbents that are novel materials for pollutant removal need high stability and reusability. However, the structural stability of the adsorbent after use is important. The FT-IR spectra after lindane adsorption (Figure 12B) indicated a change in surface functional groups, but the characteristics peaks of NAH did not change significantly. Figure 13 shows the XRD pattern of NAH after lindane adsorption and SDS modification to prove the structural stability of the adsorbent.

**Figure 14:**
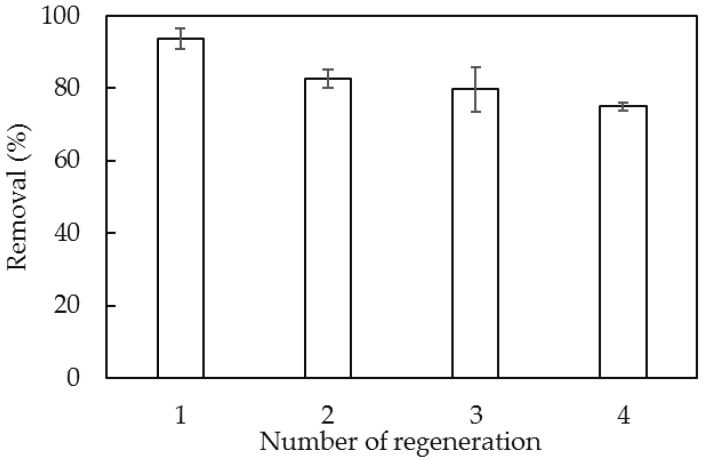
Removal efficiency of lindane using SMNAH after three regenerations. Error bars show standard deviation of three replicates.

Figure 13 indicates that the Al(OH)_3_ bayerite phase did not change, although the intensities of all peaks were decreased. The decrease of the intensity for all peaks was due to the influence of vigorous shaking and the surface rub-off and material breakup. The surface modification of SDS also affected the adsorbent. However, all characterized peaks for the bayerite phase still occurred, indicating that the structure of NAH still remained after lindane adsorption and SDS modification. In addition, the removal efficiency after some regeneration using SMNAH for lindane removal needs to be determined. Figure 14 shows the lindane removal using SMNAH after four regenerations. Although the lindane removal decreased, the efficiency was still about 75% after reuse of adsorbent. The error bars show that the standard deviations of replicates were also small, indicating that SMNAH is not only a high-performance adsorbent but also reusable for lindane removal from aqueous solution.

Surfactant-modified alumina (SMA) is now a famous adsorbent for removal of many hydrophilic pollutants; namely, heavy metal ions [50,51], ammonium ion [24], cationic dyes [21,52], antibiotics [25] and phenol [53]. On the other hand, SMNAH used in present study has not been investigated to remove highly hydrophobic pesticides, such as lindan. The advantages of SMNAH-containing admicelles is that alkyl bilayer cores can significantly enhance the hydrophobicity. The reproducibility and reusability, stability and recycling of SMNAH are suitable for not only lindane but also other hydrophobic POPs. This work is of great importance to environmental matters, such as green development, and sustainable materials, such as SMNAH.

## 4. Conclusions

This paper reported the first comprehensive study on lidane removal using synthesized nano-aluminum hydroxide (NAH) with surface modification by bilayer formation of SDS admicelles. The structural phase of Al(OH)_3_ bayerite was confirmed by XRD. The particle size of NAH was about 30 nm and the specific surface area was 154.4 m^2^/g. The surface functional groups of Al–OH and Al–O bonds of NAH were confirmed by FT-IR spectroscopy, while the charging behavior of NAH at different pH levels was examined by zeta potential. The lindane removal using SDS modified nano-aluminum hydroxide nanoparticles (SMNAH) reached 93.68%, while the adsorption capacity increased 22.8 times when using SMNAH. The adsorptive removal conditions were systematically investigated and found to be pH 6, adsorption time 60 min, adsorbent dosage 25 mg/mL and ionic strength 10 mM NaCl. Lindane removal using SMNAH was still about 75% after four re-cycles. The adsorption isotherms of lindane onto SMNAH at two salt concentrations were reasonably fitted by a two-step adsorption model. SMNAH achieved a much higher removal efficiency and adsorption capacity of lindane than other adsorbents. The structural stability of NAH after SDS modification and lindane adsorption was also proven. The SDS admicelles bilayer on NAH induced a significant increase in the hydrophobic interaction, which was the main mechanism for lindane adsorption onto SMNAH.

## Figures and Tables

**Table 1:** The parameters of GC-ECD for lindane determination.

Parameters	GC-ECD
Injector temperature	250 °C
Injection type	Splitless
Injection volume	2 μL
Carries gas flow rate	Nitrogen (99.99%), 1.2 mL·min^−1^
Make-up gas	Nitrogen (99.99%), 25 mL·min^−1^
Oven temperature	160 °C (hold 2 min), at 5 °C /min to 280 °C (4 min). Total running time: 18 min.
Detector temperature	300 °C

**Table 2:** The fit parameters for lindane adsorption isotherms onto SDS modified nano-aluminum hydroxide (SMNAH).

C_NaCl_ (mM)	Γ (µg/g)	*k*_1_ (10^3^ g/mg)	*k*_2_ (10^3^ g/mg)^n−1^	*n*
10	325	5.0	8.5	2.1
1	320	5.0	8.0	2.1

**Table 3:** Adsorption capacity and removal efficiency of surfactant-modified nano aluminum hydroxide (SMNAH) and other absorbents for lindane removal.

Adsorbent	Adsorption Capacity (µg/g)	Removal Efficiency (%)	References
Bagasse fly ash	2.51	NI	[1]
Fungal biosorbent	NI	82.75	[10]
Biomimetic fat cell	NI	72.03	[45]
Prepolymer	NI	15.65	[45]
Clinoptilolite rock	5.6	68.0	[46]
Soil slurry	35.3	NI	[47]
Aquifer sand	NI	73.2	[48]
Pine bark	3.14	83.51	[49]
Surfactant-modified nano-aluminum hydroxide (SMNAH)	325.0	93.68	This work

NI: no information.
